# Integrating Kata Training into School Education: Effects on Sustained Attention and Cognitive Performance in 8–9-Year-Old Children

**DOI:** 10.3390/children12020208

**Published:** 2025-02-10

**Authors:** Fuat Gökdere, Erdem Uylas, Fatih Çatıkkaş, Erkan Günay, Halil İbrahim Ceylan, Murat Özgören

**Affiliations:** 1 Institute of Graduate Studies, Manisa Celal Bayar University, 45140 Manisa, Türkiye; 231386001@ogr.cbu.edu.tr; 2 Department of Physical Education and Sports, Dokuz Eylul University, 35330 Izmir, Türkiye; uylas.erdem@ogr.deu.edu.tr; 3 Department of Coaching Education, Manisa Celal Bayar University, 45040 Manisa, Türkiye; fatih.catikkas@cbu.edu.tr; 4 Department of Physical Education of Sports Teaching, Faculty of Sports Sciences, Atatürk University, 25240 Erzurum, Türkiye; 5 Brain and Conscious States Research Centre, Department of Neuroscience, Institute of Graduate Studies, Department of Biophysics Faculty of Medicine, Near East University/KKTC, via Mersin 10, 99138 Nicosia, Türkiye; murat.ozgoren@neu.edu.tr

**Keywords:** sustain attention, pre-school children, cognitive processing speed, kata exercise, academic achievement

## Abstract

(1) Background: The ability to sustain attention in primary school children aged 8–9 years plays a critical role in maintaining focus for extended periods, enabling them to comprehend and integrate large amounts of information. Enhancing sustained attention during this formative stage significantly improves a child’s capacity to acquire and consolidate new skills and knowledge, laying a strong foundation for academic and cognitive development. (2) Objectives: This study aimed to assess the effect of an 8-week kata training program on attention and its components in 8–9-year-old school-age children, a critical developmental period for attention. (3) Methods: After excluding the participants who gave a low number of correct answers in the pre-test or created a ceiling effect, 43 participants, aged 9.12 ± 0.40 years, were included in this study. This study, conducted during the academic term, included three measurement phases and a familiarization session. Attention parameters were evaluated using the Bourdon–Vos Test, and participants were categorized into Low Performers (LP) and High Performers (HP) based on pre-test scores. The intervention group (INT) underwent kata training thrice weekly for 8 weeks, while the control group (CON) followed their regular activities. Post-training, attention parameters were reassessed using the Bourdon–Vos Test. (4) Results: In the post-intervention analysis, significant improvements in the number of correct responses were observed in both the LP (*p* < 0.001, Cohen’s d = −1.333) and HP (*p* = 0.001, Cohen’s d = −1.644) groups within the INT group. In the CON group, significant improvement was observed only in the HP group (*p* = 0.031, Cohen’s d = −0.948). Regarding attention processing speed, significant pre-post improvements were found exclusively in the INT group (*p* < 0.001). Block-wise analysis revealed significant differences only in Block 1 of the CON group (*p* = 0.011, Cohen’s d = −0.522). However, in the INT group, significant improvements were observed in both Block 1 (*p* < 0.001, Cohen’s d = −1.200) and Block 2 (*p* = 0.004, Cohen’s d = −0.678). (5) Conclusions: The findings of this study highlight the effectiveness of an 8-week kata training program in enhancing sustained attention and cognitive processing speed among 8–9-year-old children, particularly in low-performing groups. This suggests that integrating structured kata-based motor and cognitive activities into school curricula can serve as a promising strategy for addressing attention deficits and promoting cognitive development during this critical developmental period. Future studies should examine the long-term effects of kata training on attention and related cognitive functions, such as working memory and executive control. Investigating neurophysiological mechanisms through neuroimaging and including diverse age groups with larger samples could further validate these findings.

## 1. Introduction

Especially in primary school children, academic performance, multi-tasking, short- and long-term memory, recall, and sustained attention are the leading cognitive skills [1]. Recent systematic reviews and meta-analyses in the literature have provided evidence of the long-term effects of exercise programs on children’s cognitive performance [2]. These studies have observed activity duration-dependent changes during or immediately after structured physical activity [2,3,4]. Although the acute effects of exercise on cognitive performance, such as attention, inhibition, and working memory, are generally known [5,6] the findings of recent studies indicate that only moderate-intensity exercise lasting 10 min or more improves cognitive performance [6]. Previous findings particularly support the positive effects on the development of selective attention [7,8,9,10,11]. Positive effects on cognitive performance are observed in primary school children who perform exercise sessions lasting 30–60 min, 3–5 days a week [12,13]. Neurobiological, psychological, and human performance-related theories illustrate these effects. The changes that increase brain volume, such as increased availability of growth factors, increased developmental growth, the formation of new blood vessels, and the development of neurons, may increase the efficiency of neural networks and, consequently, cognitive performance [14,15]. Practices applied for a relatively sustained period (6–8 weeks) contribute to an increase in cognitive performance. Exercises such as karate and gymnastics, which involve repetitive tasks, are known to enhance cognitive performance to a greater degree [16,17].

Kata is the presentation of defense and attack techniques in the regular form in a predetermined sequence [18]. These contain special skills such as reaction speed, strength, continuity, balance, and coordination. Kata, which is an imaginary fight method of karate usually applied in childhood, includes an aerobic-based exercise design that focuses on technical development [19]. The aim is known to improve the ability to learn techniques and correct posture and breathing techniques [20]. During the exercise, participants are expected to perform the memorized kata series in a certain flow and order and to successfully perform the figures that distinguish the performance with a high level of difficulty. During this process, cognitive skills support the realization of performance under high physical load. Research on a limited number of kata revealed that heart rate during kata was 179 ± 11.55 beats per minute (bpm), and oxygen consumption level was between 36.8 ± 5.4 mL/min 40 [21]. During an effort, complex workloads that use both aerobic and anaerobic energy pathways and regularly switch between dynamic and static (equilibrium) phases naturally take this information into account. In kata, not confusing the sequence of movements during the series and performing them at the correct flow rate emphasizes the need for cognitively sustainable attention [22].

Sustained attention, or vigilance, refers to the ability to sustain attention over a long period. The capacity to sustain attention determines the child’s capacity to maintain concentration for long periods to understand and integrate large amounts of information. Therefore, disturbances in sustained attention negatively affect the child’s ability to acquire and integrate new skills and knowledge. In age-specific comparative studies, it has been reported that attention develops rapidly between the ages of 8 and 10 years and stagnates between the ages of 10 and 13 years [23]. However, another study reported a steady improvement in sustained attention skills between the ages of 8 and 16 years, but the magnitude of gains decreased between approximately 10 and 11 years [24]. While these studies provide a better understanding of attentional development for children aged eight years and older, research into the relative sustained attention capacities of children, particularly in the early years of school, is ongoing. It is understood that the information presented here indicates that 8–9 years of age is a ‘critical period for attention development’. The main motivation for this study is that the research literature on attention-exercise-child-related specific to primary school children aged 8–9 years is less developed [25].

In light of the information provided above, this study was designed to examine the cognitive changes resulting from an 8-week kata training program in primary school children. The hypotheses of this study were as follows: (i) An 8-week kata training program integrated into academic education improves attention performance in 8–9-year-old school-age children; (ii) compared to the group with high attention performance, participants with lower attention performance demonstrate greater improvements in sustained attention performance after kata training compared to the control group with similar attention levels; and (iii) cognitive processing speed improves faster in the intervention group compared to the control group.

## 2. Materials and Methods

### 2.1. Participants

This study included 60 participants [30: intervention (INT), 30: control (CON)]. The G-Power 3.1.9.4 software (Heinrich Heine University Düsseldorf, Düsseldorf, Germany) was used to a priori determine the required sample size (ANOVA: repeated measures, between factors). An effect size f = 0.50 (i.e., effect size d has been converted to f via Walter et al. (1998) was used [26], with an alpha (error) rate of 5% and a power of 95% to estimate the required sample size for repeated measures ANOVA with two different measures of the two groups. The determination in G-Power yielded a total sample size of 42 participants. To prevent possible data loss, a total of 60 participants were included in this study. All participants and their parents filled out the informed consent form, and the study documented their voluntary participation. The Manisa Celal Bayar University Health Sciences Ethics Committee approved this study with a decision dated 27 March 2024, and number 20.478.486/2325. The Declaration of Helsinki guided the conduct of this study.

### 2.2. Determination of Participant Groups

**Figure 1:**
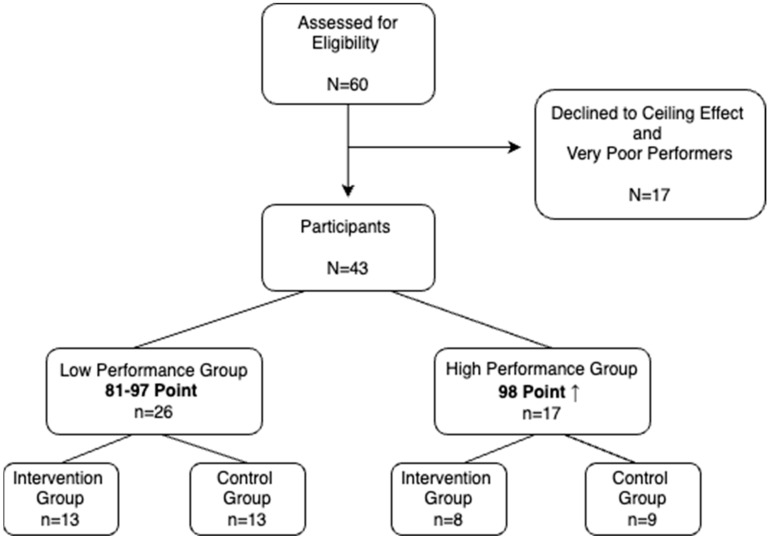
Participation flow chart.

This study included 60 participants [30: intervention (INT), 30: control (CON)] who qualified for inclusion (N = 60). Those who performed very poorly and with a ceiling effect in the pre-test accuracy were excluded from the study (N = 17). The remaining 43 participants were divided into two groups according to their performance in the pre-test number of correct answers. Initially, 60 children met the inclusion criteria, but those who performed very poorly and those with a ceiling effect in the pre-test accuracy were excluded from the study (N = 17). This division was performed by calculating the median values of the number of correct answers given by all participants [27,28]. According to the median values, those with 81–97 correct values formed the ‘Low-Performance Group (LP)’, and those with 98 correct values and above formed the ‘High-Performance Group (HP)’. LP included 13 INT and 13 CON group participants. The HP group consisted of 8 INT and 9 CON participants. The flow chart in Figure 1 illustrates this structure.

### 2.3. Study Design

**Figure 2:**
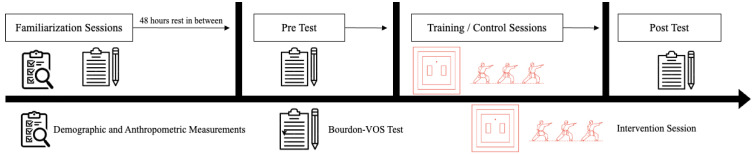
Study design outlining the process, including familiarization and demographic/anthropometric data collection, pre-test Bourdon–Vos Test assessment, Kata intervention (for the intervention group only), and post-test Bourdon–Vos Test assessment.

This study was designed as a randomized controlled trial with intervention (INT) and control (CON) groups. The study included three sessions, including a familiarization session, a pre-test, an intervention kata/control, and a post-test session. The practice, pre-test, and post-test sessions were completed in approximately 20 min. Sessions and exercises were conducted in a similar temperature and humidity environment. In the practice session of this study, participants were introduced to the three parts of the Bourdon–Vos Test, and the letters to be followed were explained. They were asked to complete one part in 2 min. The test was repeated in two practices to eliminate any learning effect. The intervention and control groups were then determined by the random distribution method, and participants were distributed among the groups. The tests were conducted at similar times of the day in a bright and quiet environment. In the pre- and post-test sessions, all participants were asked to complete the 3 sections, each of which was completed in 2 min, for a total of 6 min. Afterwards, the 8-week kata training was started. The experimental group received kata training three times a week for 40 min for 8 weeks. The control group continued their daily activities for 8 weeks and received no other training besides their academic education. Since both groups were in the same school, they were subjected to the same academic program. After the 8-week period, the test was repeated with the same procedures. Figure 2 presents the study design and the processes of all sessions.

#### 2.3.1. Familiarization Session

After explaining the experimental protocol of the study, we collected demographic information in the first stage. Anthropometric measurements were then taken. After participants learned the Bourdon–Vos Test, the three parts of the test and the letters to be followed were explained, and they were asked to perform a part for 2 min. After two repetitions of the test, the learning effect was eliminated.

#### 2.3.2. Anthropometric Measurements

The same person measured the participants’ height using a manual, hand-held tape measure. When the participants stood upright, barefoot, with their backs flat against a wall, their height was measured by determining the top of their heads and recorded in centimeters. Body mass was measured using an electronic scale. The participants stood barefoot and upright on the electronic scale until the results appeared. The data obtained were recorded in kilograms [29,30,31].

#### 2.3.3. Bourdon–Vos Test

The Burdon–Vos Test was administered in a non-digital form with paper and pencil. There is no age limit for the test application. However, for the letter form, children must have attained the ability to identify letters. The Bourdon–Vos Test is a measurement tool frequently used in 8–9-year-old children [32]. They complete the letter form of the Bourdon–Vos Test, which is designed to evaluate their ability to maintain uninterrupted attention. The test battery consists of 660 letters in total. The test will take 2 min per section, totaling 6 min. Essentially, the task required the participants to locate and identify the letters “a”, “b”, “d”, and “g” within the written letter text. The evaluation process will consider the number of correct and incorrect answers. Each correct answer is accepted as one point. The highest score that can be obtained from the test was determined to be 118. The increase in the individual’s score will indicate a higher level of attention. The test determined and recorded the individuals’ performances based on the scores they collected [33].

### 2.4. Kata Training Program

In this study, an 8-week (24 sessions) karate-do kata basic training was implemented as a controlled basic training process outside the school curriculum and physical education classes. The kata training program involved three sessions per week, with each session lasting 40 min. The training sessions were led by an instructor–researcher holding a 5th Dan and a 2nd Level National Coaching Certificate, who was also the co-researcher of this study. The main content of the training was based on the ‘Pinan Nidan Kata Technique’, which included movements such as stances, blocks, kicks, and kick techniques. Each training session consisted of 10 min of warm-up (kata-specific movement patterns and flexibility), 25 min of kata basic movements series, and 5 min of stretching exercises. In the 25-min main phase, the difficulty and repetition of the movements were increased as the weeks progressed. The kata movement forms in the study were selected from the forms used in the literature [20,34]

### 2.5. Statistical Analysis

The mean and standard deviation (SD) values of the Bourdon–Vos Test were calculated for all conditions (INT and CON), performance groups (HP and LP), and temporal durations (pre and post). The values obtained were analyzed using the JASP (Just Another Statistical Program, version 0.16.4) statistical package [35]. The normal distribution of the data was determined by the Shapiro–Wilk analysis. The variation values of the normally distributed data in the INT and CON conditions were analyzed using a paired sample *t*-test. Performance groups (HP and LP) were also compared using a paired sample *t*-test. Effect sizes of the obtained data were determined using Cohen’s d, and comparisons were made. In all statistical analyses, the significance level was determined as less than 0.05 (*p* < 0.05) [35].

Additionally, we calculated the participants’ ‘Accuracy Rate’ by dividing the total number of correct answers by the total number of participants’ answers, resulting in an accuracy rate of [Accuracy rate = Total number of correct answers (118)/Number of participants’ answers] [36]. The remoteness to the maximum score value was recorded as the distance to the maximum number of responses. In other words, the maximum number of responses (118) was accepted as 100%. The distance to the maximum score was calculated by subtracting the accuracy rate from this percentage value [Remoteness = Maximum score – Accuracy rate] [37]. Processing speed was obtained by dividing the total response time given to the participants during the test by the total correct responses [Processing speed = Total time/Correct answers] [38]. This value indicated how much time the participants spent on a response in seconds.

## 3. Results

Table 1 provides descriptive and anthropometric data for 43 participants. The mean age for 23 male and 20 female participants was 9.12 ± 0.40 years, height 131.57 ± 6.48 cm, and body mass 30.59 ± 6 kg. The mean age of the 7 male and 14 female participants in the intervention groups (HP and LP) was 9.12 ± 0.35 years, height 134.00 ± 7.38 cm (cm.), and body mass 31.96 ± 8.45 kg (kg). The mean age of the 16 male and 16 female participants in the control groups (HP and LP) was 9.11 ± 0.32 years, height 131.96 ± 8.45 cm, and body mass 28.63 ± 6.05 kg. The anthropometric and demographic characteristics of both groups were similar.

**Figure 3:**
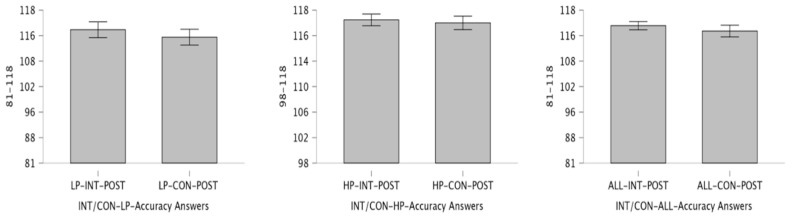
Post-session correct response change counts analysis between performance groups. The left panel (LP), center panel (HP), and right panel (ALL) groups are shown in the figure. Asterisks indicate the level of statistical significance. Each panel contains three blocks of 6 min each in which the Bourdon–Vos Test was performed.

**Figure 4:**
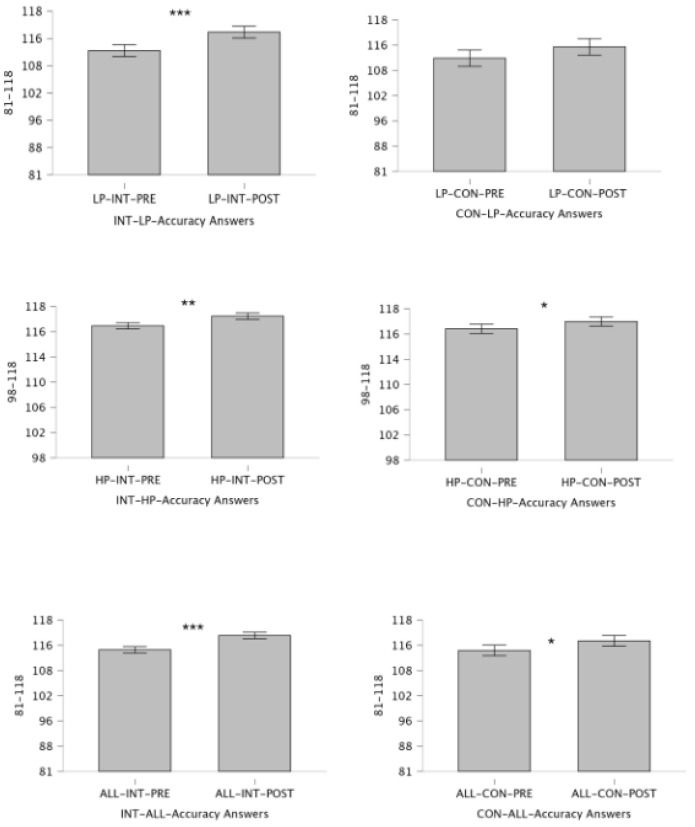
Performance groups and analysis of accuracy response number of changes before and after conditions. The figure displays the upper panel (LP), middle panel (HP), and bottom panel (ALL) groups. Asterisks indicate statistical significance level: * *p* < 0.05, ** *p* < 0.01, *** *p* < 0.001. Each panel includes three blocks, each lasting 6 min, where the Bourdon–Vos Test is administered.

The pre-test Bourdon–Vos Test accuracy response results between groups are presented in Table 2. No statistically significant difference was found in the LP, HP, and ALL groups of INT and CON participants (respectively: LP pre = *p*: 0.719, Cohen’s d = 0.102; LP post: *p* = 0.173, Cohen’s d: 0.402; HP pre = *p*: 0.531, Cohen’s d: 0.233; HP post = *p*: 0.457, Cohen’s d: 0.278; ALL pre = *p* = 0.782, Cohen’s d: 0.066; ALL post = *p*: 0.196, Cohen’s d: 0.318). Post session correct response change are presented in Figure 3 and Performance groups and analysis of accuracy response number of changes before and after conditions presented in Figure 4.

**Figure 5:**
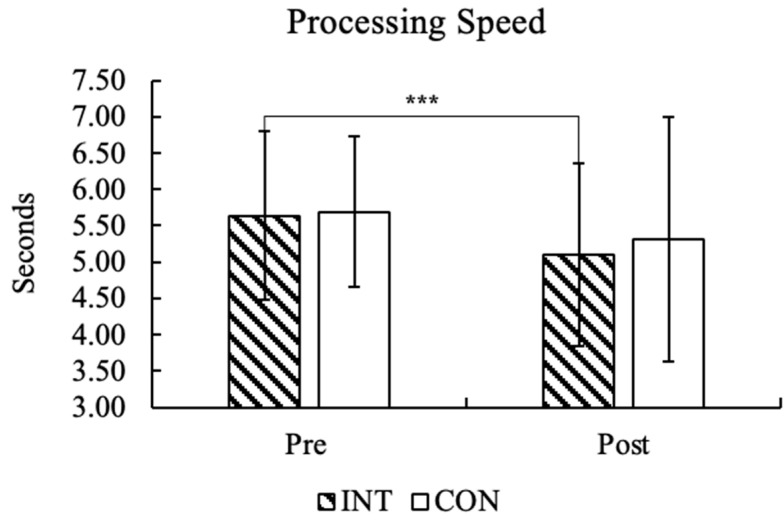
Processing speed values in the pre-post Bourdon–Vos Test results of all groups are demonstrated by vertical bars, and significance is marked with an asterisk, *** *p* < 0.001.

The LP and ALL groups of INT participants showed a statistically significant difference (LP = *p*: 0.001, Cohen’s d = 1.149, ALL = *p*: 0.55, Cohen’s d = 0.487), while the HP group showed no significant difference (*p* = 0.297, Cohen’s d = 0.371). The HP group of CON participants showed a statistically significant difference (HP = *p*: 0.039, Cohen’s d = −0.894). In contrast, the LP and ALL groups showed no significant difference (LP = *p*: 0.118, Cohen’s d = 0.468, and ALL = *p*: 0.55, Cohen’s d = 0.487). Pre-post processing speed results of the INT and CON groups are presented in Figure 5.

**Figure 6:**
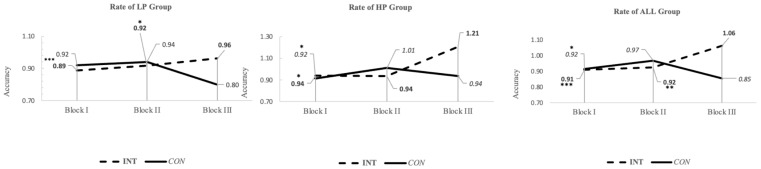
Block analysis on the rate of accuracy response changes between performance groups and conditions. The **left** panel (LP), **mid** panel (HP), and **right** panel (ALL) groups are shown. The dashed line indicates the intervention, and the solid line refers to the control group. The vertical axis presents an accuracy index. Each panel incorporates three blocks of time for the administration of the Bourdon–Vos Test, with 2 min each. * *p* < 0.05, ** *p* < 0.01, *** *p* < 0.001.

The Bourdon–Vos Test block analysis results of the within-group pre- and post-test are presented in Table 3. A statistically significant difference was found in the LP Block-I, HP Block-I, and ALL groups Block I and II of INT participants (respectively: LP Block-I = *p* < 0.001, Cohen’s d = −1.431; HP Block-I = *p*: 0.024, Cohen’s d = −0.922; ALL Block-I = *p* < 0.001, Cohen’s d = −1.200; ALL Block-II = *p*: 0.004, Cohen’s d = −0.678). The HP Block-I and ALL groups Block-I of CON participants showed statistically significant differences (HP Block-I = *p*: 0.020, Cohen’s d = −1.062; and ALL Block-I = *p*: 0.011, Cohen’s d = −0.522). INT and CON for other block times, and all performance group parameters, were not statistically significant. Additionally, the block analysis on the rate of accuracy answer changes between performance groups and conditions is presented in Figure 6.

The changes observed in both the intervention and control groups can be summarized as follows: Participants in the intervention group, regardless of their performance level (HP or LP), were able to maintain their focus and continue engaging with the task for the entire duration. This is particularly evident during Block III in each group (Figure 4). This ability to sustain focused attention holds potential benefits for children’s future activities, from academic achievement to sports performance.

## 4. Discussion

This study aimed to investigate the effects of 8 weeks of kata training on attention and its derivatives in 8–9-year-old primary school children. The main finding of this study was that regular kata training significantly improved sustained attention characteristics and attention-related processing speed in these children. The main hypothesis of this study was confirmed.

A limited number of studies examine the relationship between long-term physical activity and attention. De Greeff et al. reported an improvement in executive memory functions, attention, and academic performance of school children aged 6–12 years after aerobic, anaerobic, and cognitive-based physical activity interventions, respectively [9]. Our study is among the rare studies evaluating the responses of sustainable attention performance specific to a sports branch in the 8–9 age range. Our study results showed that 8 weeks of regular kata training positively affected the performance in the preadolescent age group, which is known to be the critical period in sustained attention performance (total and block 3).

The literature on the topic demonstrates that martial arts practices improve executive functions, including attention, self-discipline, and inhibitory control in children aged 4–12 years [39]. On the other hand, structured physical activities, especially those requiring dual-task processing (physical and mental engagement), have been shown to improve general cognitive performance, including attentional skills [40]. Furthermore, interventions involving martial arts have shown improved behavioral regulation and academic outcomes in children with ADHD, highlighting martial arts as a potential attention-enhancing tool [41].

Our study highlights the cognitive benefits of 8-week kata training for sustained attention and processing speed in children, focusing on its structured approach combining physical precision with cognitive engagement. Attention is a critical skill for learning and daily functioning and consists of various components, including sustained attention (the ability to focus over time), selective attention (focusing on relevant tasks while ignoring distractions), and executive control (regulating and shifting focus as needed) [24]. Kata training requires children to memorize and execute choreographed movements, promoting sustained attention by encouraging prolonged focus while simultaneously enhancing selective attention by filtering distractions and maintaining concentration on precise movements. This dual engagement of motor actions and memory activates the prefrontal cortex, a brain region critical for regulating attention and cognition [42]. Techniques such as controlled breathing and mindfulness embedded in kata practice further support arousal regulation, creating an optimal state for focused attention. Research has shown that martial arts, in general, improve children’s cognitive functions, including attention, self-regulation, and decision-making [41]. Our findings suggest that kata training may have similar effects, with its structured environment and progressive skill-building boosting attention in a way that may be transferable to other areas of life.

Over time, kata training fosters attentional endurance and self-discipline, which can translate into benefits in academic tasks, sports, and other activities. The Bourdon–Vos Test results demonstrated significant improvements in processing speed in the kata training group compared to the control group. Processing speed, a fundamental cognitive skill required for efficient task performance, is critical for both academic success and daily functioning. The repetitive and progressive nature of kata movements likely strengthens neural efficiency by requiring individuals to process patterns, adjust movements, and refine execution continuously. These findings suggest that structured motor-cognitive activities, such as kata training, provide unique advantages over unstructured physical activities by integrating disciplined practice with cognitive focus.

Kata training also reinforces motivation and persistence through the mastery of sequences, encouraging children to engage in longer periods of focused activity (as illustrated in Figure 5). This combination of benefits positions kata as a promising intervention to address challenges such as attention deficits, including ADHD. Despite these promising results, further research is essential to confirm these findings and extend their implications. Larger sample sizes, longer intervention periods, and the inclusion of neuroimaging techniques to explore the underlying neural mechanisms would provide deeper insights into the long-term effects of kata training on children’s cognitive and attentional development. Incorporating kata into systematic school programs could prove valuable for integrating both motor and cognitive development, further supporting foundational skills important for academic and personal success.

Combining physical precision with cognitive engagement, kata training can help children maintain prolonged focus, regulate distractions, and strengthen neural efficiency. Schools can implement this training during physical education classes or as part of extracurricular activities to foster academic performance, attentional capacities, and self-discipline. Additionally, the structured, progressive nature of kata training may provide long-term benefits, such as improved executive functioning, which could positively influence other areas of life, including sports and daily tasks. These findings suggest that kata training is a promising intervention for addressing attention deficits and enhancing overall cognitive health in school-aged children.

## 5. Conclusions

The findings of this pilot study suggest that an 8-week kata training program can provide significant benefits in improving sustained attention performance in 8–9-year-old children, with significant improvements observed, especially in the low-performance (LP) intervention group. Furthermore, this development shows that kata training can be positioned as an alternative to prevent increasing attention deficit problems in children. The implementation of systematic programs that integrate motor and cognitive activities in the 8–9 age range, known as the period of rapid brain and peripheral myelination, may play an important role in promoting cognitive and neural development, especially in attention-related areas. These results highlight the importance of further examination of such interventions in future research to uncover the neurophysiological mechanisms driving their medium- and long-term effects. Investigating how kata training affects brain regions and connections will provide deeper insights and strengthen its potential as a valuable tool for improving cognitive and attention capacities in children.

## 6. Limitations

This study has some limitations. Only responses to a 6-min letter-based attention task were analyzed in the study. In future studies, the need to examine sustained attention performance in longer time blocks specific to 8–9 years of age emerges. In addition, although a narrow age range was considered in this study, it is thought that the number of participants was limited. Therefore, there is a need for studies with a larger group of participants, more complex attention tasks, and studies in which differences between genders are also observed.

## Figures and Tables

**Table 1:** Within-group Bourdon–Vos Test pre-test and post-test results.

Conditions	Performance	Time	Mean ± SD	*p*	Mean Difference	Cohen’s d	Accuracy Rate %	% Remoteness to Maximum Score
**INT** **Accuracy**	LP	Pre	91.07 ± 7.50	<0.001 ***	13.54	−1.333	77.18	22.82
		Post	104.61 ± 8.84				88.66	11.34
	HP	Pre	104.66 ± 4.09	0.001 **	7.67	−1.644	88.70	11.30
		Post	112.33 ± 4.41				95.20	4.80
	ALL	Pre	96.48 ± 8.85	<0.001 ***	11.32	−1.299	81.7	18.23
		Post	107.80 ± 7.91				91.36	8.64
**CON** **Accuracy**	LP	Pre	89.76 ± 7.58	0.055	9.01	−0.590	76.07	23.93
		Post	98.77 ± 15.18				83.71	16.29
	HP	Pre	104.12 ± 3.35	0.031 *	5.88	−0.948	88.24	11.76
		Post	110.00 ± 6.16				93.23	6.77
	ALL	Pre	95.94 ± 8.85	0.015 *	7.71	−0.635	81.4	18.69
		Post	103.55 ± 13.70				87.76	12.24

Note: INT: intervention groups, CON: control group, ALL: all group, HP: high-performance group, LP: low-performance group, statistical differences level: * *p* < 0.05, ** *p* <0.01 *** *p* < 0.001, %: maximum-performed (accuracy), Cohen’s d: effect size.

**Table 2:** Bourdon–Vos Test pre-test results between groups.

Performance	Conditions	Time	Mean ± SD	*p*	Cohen’s d
**LP**	INT	PRE	91.07 ± 7.50	0.719	0.102
	CON		89.76 ± 7.58		
	INT	POST	104.61 ± 8.84	0.173	0.402
	CON		98.76 ± 15.18		
**HP**	INT	PRE	104.66 ± 4.09	0.531	0.233
	CON		104.12 ± 3.35		
	INT	POST	112.33 ± 4.41	0.457	0.278
	CON		110.00 ± 6.16		
**ALL**	INT	PRE	96.48 ± 8.85	0.782	0.066
	CON		95.94 ± 9.84		
	INT	POST	107.80 ± 7.91	0.196	0.318
	CON		103.55 ± 13.70		

Note: ALL: all group, LP: low-performance group, HP: high-performance group, INT: intervention groups, CON: control group, *p*: statistical differences *p* < 0.05.

**Table 3:** Within-group block analysis Bourdon–Vos Test accuracy scores pre-test and post-test results.

Conditions	Performance	Blocks	Time	Mean ± SD	*p*	Mean Difference	Cohen’s d
**INT** **Accuracy**	LP	Block I	Pre	33.76 ± 2.65	<0.001 ***	4.31	−1.431
			Post	38.07 ± 2.01			
		Block II	Pre	30.46 ± 3.52	0.015 *	2.84	−0.790
			Post	33.30 ± 2.32			
		Block III	Pre	27.84 ± 8.72	0.064	5.39	−0.565
			Post	33.23 ± 8.37			
	HP	Block I	Pre	37.77 ± 2.43	0.024 *	2.25	−0.922
			Post	40.02 ± 1.09			
		Block II	Pre	32.88 ± 4.04	0.156	2.45	−0.522
			Post	35.33 ± 1.48			
		Block III	Pre	35.22 ± 3.45	0.820	−0.78	0.078
			Post	34.44 ± 9.01			
	ALL	Block I	Pre	35.40 ± 3.21	<0.001 ***	3.55	−1.200
			Post	38.95 ± 1.98			
		Block II	Pre	31.45 ± 3.85	0.004 **	3.64	−0.678
			Post	34.09 ± 2.20			
		Block III	Pre	30.86 ± 7.86	0.191	2.86	−0.288
			Post	33.72 ± 8.45			
**CON** **Accuracy**	LP	Block I	Pre	35.42 ± 6.31	0.096	3.5	−0.501
			Post	38 ± 92 ± 2.72			
		Block II	Pre	30.61 ± 4.64	0.098	2.92	−0.497
			Post	33.53 ± 5.39			
		Block III	Pre	23.69 ± 11.91	0.642	2.46	−0.132
			Post	26.15 ± 15.10			
	HP	Block I	Pre	35.75 ± 1.90	0.020 *	3.5	−1.062
			Post	39.25 ± 2.60			
		Block II	Pre	33.87 ± 1.88	0.919	−0.12	0.037
			Post	33.75 ± 3.01			
		Block III	Pre	34.00 ± 3.70	0.204	2.75	−0.439
			Post	36.75 ± 4.23			
	ALL	Block I	Pre	35.57 ± 5.02	0.011 *	3.47	−0.610
			Post	39.04 ± 2.61			
		Block II	Pre	31.85 ± 3.97	0.156	1.76	−0.522
			Post	33.61 ± 4.54			
		Block III	Pre	27.61 ± 10.78	0.820	2.58	0.078
			Post	30.19 ± 13.07			

Note: INT: intervention groups, CON: control group, ALL: all group, HP: high-performance group, LP: low-performance group, *p*: statistical differences *p* < 0.05, %: maximum-performed (accuracy), Cohen’s d: effect size. * *p* < 0.05, ** *p* < 0.01, *** *p* < 0.001.

## Data Availability

All collected data in the current study are available after obtaining permission from all of the authors. Written proposals can be addressed to the corresponding authors for appropriateness of use. The data are not publicly available due to privacy and ethical reasons.
